# Complete Mitochondrial Genome of the Eggplant Fruit and Shoot Borer, *Leucinodes orbonalis* Guenée (Lepidoptera: Crambidae), and Comparison with Other Pyraloid Moths

**DOI:** 10.3390/insects15040220

**Published:** 2024-03-25

**Authors:** Joshua B. Despabiladeras, Ma. Anita M. Bautista

**Affiliations:** Functional Genomics Laboratory, National Institute of Molecular Biology and Biotechnology, College of Science, University of the Philippines-Diliman, Quezon City 1101, Philippines; jbdespabiladeras@up.edu.ph

**Keywords:** eggplant fruit and shoot borer, whole genome sequencing, mitochondrial genome, pest genomics

## Abstract

**Simple Summary:**

Eggplant is an important agricultural produce in the Philippines, but consistent production is hampered by frequent infestations of the eggplant fruit and shoot borer (EFSB). The management and monitoring of EFSB infestation requires full understanding of its biology, including its evolution and adaptations. The use of genomic resources provides a starting point for understanding the biology of EFSB. However, these resources are lacking for EFSB. This study aimed to sequence and characterize the mitochondrial genome (mitogenome) of the EFSB and use the mitogenome in reconstructing the evolutionary relationships of the EFSB with respect to other members of the economically important superfamily, Pyraloidea. The annotated mitogenome of the EFSB contains the typical elements expected for a lepidopteran mitochondrion, such as gene content, gene syntenies, and nucleotide composition. Phylogenetic analyses using 72 mitogenomes from other moths robustly placed the EFSB as basal to its supposed subfamily, Spilomelinae, despite recovering the expected subfamily relationships of earlier works. The mitogenome resource for the EFSB generated here will add to the scarce genomic resources for the EFSB and be useful for future phylogenetic reconstruction of Pyraloidea and Spilomelinae.

**Abstract:**

The eggplant fruit and shoot borer (EFSB) (*Leucinodes orbonalis* Guenée) is a devastating lepidopteran pest of eggplant (*Solanum melongena* L.) in the Philippines. Management of an insect pest like the EFSB requires an understanding of its biology, evolution, and adaptations. Genomic resources provide a starting point for understanding EFSB biology, as the resources can be used for phylogenetics and population structure studies. To date, genomic resources are scarce for EFSB; thus, this study generated its complete mitochondrial genome (mitogenome). The circular mitogenome is 15,244 bp-long. It contains 37 genes, namely 13 protein-coding, 22 tRNA, and 2 rRNA genes, and has conserved noncoding regions, motifs, and gene syntenies characteristic of lepidopteran mitogenomes. Some protein-coding genes start and end with non-canonical codons. The tRNA genes exhibit a conserved cloverleaf structure, with the exception in *trnS1*. Partitioned phylogenetic analysis using 72 pyraloids generated highly supported maximum likelihood and Bayesian inference trees revealing expected basal splits between Crambidae and Pyralidae, and Spilomelinae and Pyraustinae. Spilomelinae was recovered to be paraphyletic, with the EFSB robustly placed before the split of Spilomelinae and Pyraustinae. Overall, the EFSB mitogenome resource will be useful for delineations within Spilomelinae and population structure analysis.

## 1. Introduction

The mitochondrion is a fundamental, energy-generating organelle responsible for oxidative phosphorylation [1,2]. Mitochondria are believed to have originated from the endosymbiosis of an ancestral Archaeal prokaryote with an alphaproteobacterium [3]. The energy-generating capacity of the mitochondria is fundamental to life that almost all extant eukaryotes contain fully functional or derivative forms of mitochondria [4], with the only exception to date being the flagellated excavates, *Monocercomonoides* [5]. Although most of the mitochondrial proteins are encoded by the nuclear genome, most mitochondria preserve a highly reduced genome encoding 13 protein-coding genes important for the electron transport chain and respiratory function. Mitochondrial genomes also contain a self-sufficient translation machinery consisting of a complete set of 22 tRNAs and 2 rRNAs [6]. Some mitochondrial genes (e.g., cytochrome oxidase I (COI) subunit) are already widely established for use in phylogenetics [7]. Animal mitogenomes have conserved gene content and maternal inheritance, properties that enable extensive use not only in phylogenetics but also in population genetics [8], diagnostics and species delineation [9], and molecular evolution [10,11]. Moreover, the use of multi-gene datasets in phylogenetics has additional advantages such as reducing sampling error, making it the modern standard in inferring species relationships between organisms [12,13]. 

The eggplant fruit and shoot borer (EFSB), *Leucinodes orbonalis* Guenée, is a devastating crambid pest of the eggplant, *Solanum melongena* L. The eggplant is one of the top agricultural products of the Philippines, but consistent production is hampered by frequent EFSB infestations [14]. EFSB is a member of the species-rich lepidopteran superfamily Pyraloidea, which contains at least 15,500 genera [15]. The superfamily is ecologically diverse and economically significant, having numerous major crop pests [16]. Within Pyraloidea, the EFSB is a member of the largest subfamily, Spilomelinae [17]. Obtaining reference mitogenomes for this superfamily can aid in pest monitoring and management schemes since mitochondrial genes have been used for population structure determination [18], and the development of possible control strategies such as the Trojan female [19]. Additionally, as previously mentioned, mitogenomes are useful in establishing evolutionary relationships between organisms. Reliable phylogenies are an important tool that could help understand, for instance, how host-plant ranges evolve within the family [16]. Systematics on Spilomelinae has been historically difficult due to morphological heterogeneity, but significant progress has been made with the help of mitochondrial and nuclear markers [17,20]. The use of whole mitogenomes could help resolve the systematics of Spilomelinae. However, as of 13 March 2024, only 23 whole mitochondrial sequences of Spilomelinae are on the RefSeq database. Thus, adding more mitogenome sequences and representatives could improve resolution and taxonomic delineation in this economically important subfamily. 

The continued development of high-throughput sequencing (HTS) technologies has increased accessibility for most laboratories, leading to significant developments in phylogenetics, population genetics, barcoding, and biodiversity studies. Philippine EFSB research could also benefit from genomic resources produced by HTS, which can significantly impact the implementation of pest management. Genetic resources (mitochondrial COI) and morphological traits have been used by Sagarbarria et al. (2018) to study the population structure of EFSB in the Philippines [18]. The authors concluded that a single, widespread haplotype predominates Philippine EFSB populations. However, the authors are also aware of the limitations of using COI as the sole genetic marker for population genetic analysis and have recommended the use of other markers. The analysis of whole mitogenomes is attractive since they represent a compromise between single markers and whole genome analysis. The conserved properties of animal mitogenomes, the advantage of using multi-gene datasets, and the continued ease of acquisition would provide a promising strategy for species identification and population genetics using whole mitogenomes [21]. Whole mitogenomes were successfully used to determine the population structure of the Chinese seabass (*Lateolabrax maculatus*) in China [22] and *Mansonia* mosquitoes in Brazil [23]. Here, we present a report on the fully annotated sequence of the EFSB mitochondrial genome from short Illumina sequencing reads and phylogenetic reconstruction of Pyraloidea using whole mitogenomes. The EFSB mitogenome presented here will contribute to the genetic resources of EFSB and be useful in future population studies of EFSB in the Philippines.

## 2. Materials and Methods

### 2.1. Sample Collection and Genomic DNA Extraction

Eggplants obtained from farmers from different parts of Luzon, Philippines (i.e., Tarlac, Ilocos Norte, Laguna) were individually dissected and probed for EFSB infestations. The Institute of Plant Breeding (IPB) at the College of Agriculture and Food Science, University of the Philippines Los Baños, also provided samples. EFSB samples were stored at −80 °C until extraction. 

Extraction of EFSB genomic DNA was performed using the Qiagen MagAttract HMW DNA kit (Qiagen Inc., Mckinley West Park, Taguig, Philippines). The protocol described by the manufacturer with some modifications recommended by Kingan et al. (2019) was followed: proteinase K and buffer ATL were pre-mixed, and the resulting solution was the one used for micropestle homogenization; proteinase K digestion was shortened to 2 h; all mixing steps were performed via gentle flicking; RNase A incubation time was increased to 5 min; wide-bore tips were used to transfer all solutions containing nucleic acids; and all centrifugation steps were performed at 4 °C [24]. Extracts were run on a 0.7% agarose gel for 45 min at 70 V and visualized with GelRed (Biotium Inc., Fremont, CA, USA) staining. Extracts were sent to the Philippine Genome Center for Pulse Field Gel Electrophoresis (PFGE) analysis.

### 2.2. Library Preparation and Sequencing

Suitable extracts determined by PFGE were chosen as the starting material for Illumina library preparation using the TruSeq DNA PCR-Free library prep kit (Illumina Inc., Biopolis Way, Singapore), with the IDT for Illumina TruSeq DNA UD indexes plate (Illumina Inc., Biopolis Way, Singapore) serving as the index adapters. Libraries were constructed, and quality checked using Agilent TapeStation (Agilent Technologies Inc., Yishun Ave. 7, Singapore). Libraries that were determined to have a 550 bp insert size were then sequenced on an Illumina NovaSeq 6000 system (Illumina, Biopolis Way, Singapore) using a 2 × 150 bp run for 300 cycles at the Philippine Genome Center.

### 2.3. Pre-Processing, Read Filtering, Mitogenome Assembly, and Annotation

Raw sequences were pre-processed using fastp 0.23.2 [25] to remove adapter and polyG sequences and assessed using FastQC 0.11.9 [26]. The processed sequences were then mapped to reference mitochondrial sequences using Bowtie2 2.3.5.1 [27]. Mitochondrial references were obtained from the NCBI organelle genome database and chosen based on the following criteria: close phylogenetic relationship with EFSB, tagged as a complete reference by NCBI, and must be reported or part of a published material. Following said criteria, the following mitochondrial genomes were chosen: the *Glyphodes quadrimaculalis* mitogenome, NC_022699.1 [28]; *Maruca testulalis* mitogenome, NC_024283.1 [29]; and *Omiodes indicata* mitogenome, NC_039177.1 [30]. Mapped sequences were then extracted and used as the starting input for assembly using metaSPAdes 3.15.4 [31] packaged within MitoZ 3.4 [32], with k-mer lengths of 21, 33, 55, and 77. Downstream annotation of the mitochondrial genes was performed using the annotation module of MitoZ. An additional annotation was performed using MITOS 2.0.8 [33] to confirm the annotations performed by MitoZ, determine the position of the control region, and determine the structures of the tRNAs and rRNAs. Secondary structures were verified using tRNAscan-SE 2.0.9 [34], and subsequently drawn using the StructureEditor 1.0 packaged within the RNAstructure 6.4 software package [35]. The final annotations were also manually checked to determine if the gene boundaries were consistent. Coverage analysis was performed by mapping the reads to the assembled mitogenome using bwa 0.7.17-r1188 [36] and assessing the coverage using samtools 1.16 [37] and mosdepth 0.3.2 [38]. The final mitogenome of the EFSB has been deposited and is available in GenBank under accession number PP493058.

### 2.4. Nucleotide Sequence Composition Analysis

The nucleotide composition, codon usage, and relative synonymous codon usage (RSCU) of the EFSB mitogenome were calculated using Phylosuite 1.2.3 [39]. GC skew analysis using 100 bp windows and a 20 bp step size was performed using SkewIT [40]. GC content in 20 bp sliding windows and sequencing depth per position was calculated within MitoZ. A Circos plot summarizing gene content and organization, GC content, GC skew, and coverage across all positions was created using Circos v0.69-8 [41]. 

### 2.5. Multiple Sequence Alignment and Phylogenetic Analysis

Reference sequences of representative members of Pyraloidea were obtained from the NCBI with the following search parameters: “Pyraloidea[ORGN] AND (mitochondrion[TITL] or mitochondria[TITL]) AND 10,000:20,000[SLEN]”. The downloaded Genbank files were imported to Phylosuite [39] for sequence cleaning and standardization. Only sequences flagged as “NC” were retained for analysis. The filtered sequences were then manually checked for ambiguous bases (R, Y, N, etc.), and sequences containing such bases were removed. A total of 72 working mitogenome sequences from Pyraloidea were retained after filtering and standardization. The following subfamilies were represented: Spilomelinae, Pyraustinae, Odontinae, Acentropinae, Schoenobiinae, Glaphyriinae, and Crambinae of Crambidae; and Gallerinae, Phycitinae, Pyralinae, and Epipaschiinae of Pyralidae. The full list of organisms used for the alignment is shown in Table 1. Each of the 37 genes was extracted from the reference sequences and aligned separately using MAFFT 7.505 [42], and uninformative sites were removed using Gblocks 0.91b [43]. The cleaned alignments were then concatenated to form a linear sequence of 13,953 bp for each organism using Phylosuite [39]. Partitioned maximum likelihood and Bayesian inference phylogenetic analyses were performed using IQ-tree 2.1.2 [44,45] and MrBayes 3.2.7 [46]. For maximum likelihood phylogenetic analysis, each of the 37 genes forms 1 partition initially. Automatic model selection was performed using ModelFinder in IQ-tree, and gene partitions were automatically merged to avoid over-parametrization [47]. Branch support was assessed using the SH-aLRT test with 10,000 replicates. For Bayesian inference, Metropolis-Coupled, Markov chain Monte Carlo (MC^3^) sampling was performed to estimate the tree with the highest posterior probability. The parallel version of MrBayes [46] was used alongside the BEAGLE library [48] to speed up calculations. In brief, ModelFinder was used to find the best-of-fit models, with the -mset parameter to restrict the search to models utilized by MrBayes. Partitions were then merged, and the merged partitions and their respective prior rates, gamma shape parameter, and proportion of invariant sites were then used as inputs to MrBayes. A total of three independent MCMC runs were performed, with each run containing 1 cold and 5 hot chains. The MCMC runs were run for 10,000,000 generations, with sampling every 1000 generations. Convergence was assessed when the effective sample size (ESS) for the parameters was greater than 200, and the potential scale reduction factor (PSRF) approached 1. The first 25% of the trees were discarded as burn-in, and the BI consensus trees were built using a strict consensus rule (Allcompat). The final consensus trees for both ML and BI were then drawn and annotated using ggtree [49]. Topological concordance was assessed by plotting both trees side by side and comparing the tips.

## 3. Results and Discussion

### 3.1. EFSB Mitogenome Structure and Organization

**Figure 1:**
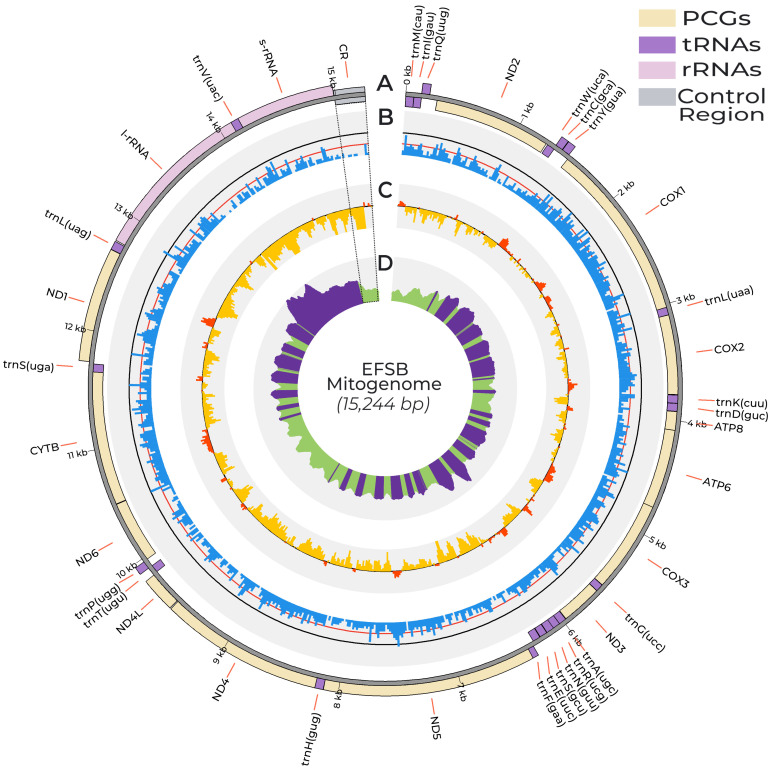
Circos plot detailing the assembled EFSB mitogenome and various statistics. (**A**) Gene organization of the EFSB mitogenome. Yellow, purple, pink, and grey boxes indicate protein-coding genes, tRNA genes, rRNA genes, and the control region, respectively. Boxes oriented inward are genes transcribed in the majority strand, while the boxes oriented outward are genes transcribed in the minority strand. (**B**) The GC content histogram was calculated in 20 bp sliding windows. Black and red lines correspond to 50% and 25% GC content, respectively. (**C**) GC-skew was calculated using a window size of 100 bp and a step size of 20 bp. Black line corresponds to 0 GC-skew value and histogram bins colored yellow and red have negative and positive GC skew values, respectively. (**D**) Coverage distribution across all positions. The EFSB mitogenome has a mean coverage of 9571×. Positions having coverage values above and below the mean coverage value are colored purple and green, respectively. The highlighted sector in dotted lines indicates the control region of the EFSB mitogenome characterized by low GC content and a switch in the sign of the GC skew values.

A total of 975,248 sequences were obtained after Bowtie2 mapping, and FastQC analysis revealed that the sequences are of high quality (Figure S1), with most of the sequences having a Phred score of 30 or above. The cleaned sequences were then used for mitogenome assembly using metaSPAdes and subsequent annotation via MitoZ and MITOS. The sequencing data obtained here yielded a minimum coverage depth value of 2215× and a maximum coverage depth value of 19,989×, yielding a mean coverage depth across all positions of 9571×. Cumulative coverage distribution obtained using mosdepth shows that 100% of the EFSB mitogenome has been covered at least 2215 times (Figure S2). The assembled mitochondrial genome has a total length of 15,244 bp and contains the 37 genes and control region expected for lepidopteran mitochondria (Figure 1).

The EFSB mitogenome contains 13 protein-coding genes, 22 tRNA genes, and 2 rRNA genes. Of the 37 genes, 23 genes (14 tRNAs and 9 PCGs) are found in the majority strand, while 14 genes (8 tRNAs, 4 PCGs, and 2 rRNAs) are found in the minority strand. The obtained mitogenome shows the typical circular double-stranded DNA structure and exhibits the conserved lepidopteran synteny of *trnM-trnI-trnQ-nad2* similarly to other crambid mitogenomes [50,51]. This synteny is characteristic of lepidopterans and differs from the ancestral insect mitogenome synteny of *trnI-trnQ-trnM*-*nad2* [52]. The EFSB mitogenome is highly AT-rich, having an AT% of 80.9%. Lepidopterans consistently have AT-rich mitogenomes with negative GC skew values (Table 1). The EFSB mitogenome is consistent with this observation, having a slightly negative AT skew of −0.01 and a negative GC-skew of −0.17. The EFSB mitogenome contains a control region with high AT content and is situated between the *s-rna* and *trnM* genes (Figure 1A,B). The control regions are also characterized by the switch in sign in the GC-skew diagrams (Figure 1C). The control region contains conserved motifs necessary for mitochondrial replication (see below) [53].

**Table 1:** Reference mitochondrial genomes chosen for composition comparison and phylogenetic analysis.

Organism	RefSeq ID	Taxonomy	AT%	AT-Skew	GC%	GC-Skew	References
*Leucinodes orbonalis*	THIS STUDY	Crambidae, Spilomelinae	80.9	−0.013	19.1	−0.172	THIS STUDY
*Acrobasis inouei*	NC_061244.1	Pyralidae, Phycitinae	80.3	−0.021	19.7	−0.219	[54]
*Aedes albopictus*	NC_006817.1	Diptera	79.6	0.008	20.5	−0.181	Direct submission
*Aglossa dimidiata*	NC_058009.1	Pyralidae, Pyralinae	79.1	−0.043	20.9	−0.226	Direct submission
*Amyelois transitella*	NC_028443.1	Pyralidae, Phycitinae	79.6	−0.048	20.4	−0.237	Direct submission
*Botyodes diniasalis*	NC_073002.1	Crambidae, Spilomelinae	80.8	−0.021	19.1	−0.182	Direct submission
*Botyodes principalis*	NC_061248.1	Crambidae, Spilomelinae	80.7	−0.014	19.3	−0.189	[54]
*Cataclysta lemnata*	NC_050323.1	Crambidae, Acentropinae	79.5	0.004	20.6	−0.214	Direct submission
*Cathayia obliquella*	NC_053657.1	Pyralidae, Gallerinae	80.6	−0.037	19.4	−0.225	[55]
*Chilo sacchariphagus*	NC_029716.1	Crambidae, Crambinae	81	−0.012	19.1	−0.246	Direct submission
*Chilo suppressalis*	NC_015612.1	Crambidae, Crambinae	80.6	0.008	19.3	−0.235	[56]
*Cnaphalocrocis* *medinalis*	NC_015985.1	Crambidae, Pyraustinae	82	−0.015	18	−0.175	[56]
*Cnaphalocrocis* *patnalis*	NC_060868.1	Crambidae, Pyraustinae	81.8	−0.022	18.2	−0.165	Direct submission
*Conogethes* *punctiferalis*	NC_021389.1	Crambidae, Spilomelinae	80.6	−0.025	19.4	−0.207	[57]
**Organism**	**RefSeq ID**	**Taxonomy**	**AT%**	**AT-Skew**	**GC%**	**GC-Skew**	**References**
*Crambus perlellus*	NC_061606.1	Crambidae, Crambinae	81.3	−0.008	18.7	−0.197	Direct submission
*Culex* *quinquefasciatus*	NC_014574.1	Diptera	78	0.007	22	−0.173	[58]
*Cydalima* *perspectalis*	NC_042150.1	Crambidae, Spilomelinae	80.9	−0.016	19.1	−0.193	[59]
*Dausara* *latiterminalis*	NC_056799.1	Crambidae, Odontinae	80.5	−0.003	19.5	−0.201	[60]
*Diatraea saccharalis*	NC_013274.1	Crambidae, Crambinae	80.1	0.021	20	−0.258	[61]
*Dioryctria rubella*	NC_061242.1	Pyralidae, Phycitinae	79.8	−0.023	20.1	−0.233	[54]
*Drosophila* *melanogaster*	NC_024511.2	Diptera	82.2	0.016	17.8	−0.147	Direct submission
*Dusungwua* *basinigra*	NC_061240.1	Pyralidae, Phycitinae	80	−0.014	20	−0.213	[54]
*Elophila* *interruptalis*	NC_021756.1	Crambidae, Acentropinae	80.3	−0.011	19.7	−0.229	[50]
*Elophila turbata*	NC_068592.1	Crambidae, Acentropinae	81.2	−0.005	18.9	−0.225	Direct submission
*Endotricha consocia*	NC_037501.1	Pyralidae, Pyralinae	79.7	−0.039	20.2	−0.226	[62]
*Endotricha* *kuznetzovi*	NC_061642.1	Pyralidae, Pyralinae	80.7	−0.033	19.2	−0.206	Direct submission
*Ephestia elutella*	NC_039716.1	Pyralidae, Phycitinae	80.7	−0.043	19.4	−0.217	[63]
*Ephestia kuehniella*	NC_022476.1	Pyralidae, Phycitinae	79.7	−0.049	20.2	−0.234	[64]
*Evergestis extimalis*	NC_071781.1	Crambidae, Glaphyriinae	80.7	−0.018	19.3	−0.168	Direct submission
*Evergestis junctalis*	NC_030509.1	Crambidae, Glaphyriinae	81	−0.015	19	−0.168	Direct submission
*Galleria mellonella*	NC_028532.1	Pyralidae, Gallerinae	80.4	−0.039	19.6	−0.237	Direct submission
*Glyphodes pyloalis*	NC_025933.1	Crambidae, Spilomelinae	80.7	−0.016	19.3	−0.194	Direct submission
*Glyphodes* *quadrimaculalis*	NC_022699.1	Crambidae, Spilomelinae	80.8	−0.007	19.2	−0.192	[28]
*Heortia vitessoides*	NC_056800.1	Crambidae, Odontinae	80.6	−0.012	19.4	−0.172	[60]
*Hypsopygia regina*	NC_030508.1	Pyralidae, Pyralinae	78.7	−0.037	21.3	−0.228	Direct submission
*Lamoria adaptella*	NC_062173.1	Pyralidae, Gallerinae	80.1	−0.012	19.9	−0.24	Direct submission
*Lista haraldusalis*	NC_024535.1	Pyralidae, Epipaschiinae	81.5	−0.007	18.5	−0.171	[65]
*Loxostege sticticalis*	NC_027174.1	Crambidae, Pyraustinae	80.8	0.002	19.2	−0.191	Direct submission
*Maruca testulalis*	NC_024283.1	Crambidae, Spilomelinae	80.8	−0.005	19.2	−0.171	[29]
*Maruca vitrata*	NC_024099.1	Crambidae, Spilomelinae	80.7	−0.002	19.3	−0.172	Direct submission
*Meroptera pravella*	NC_035242.1	Pyralidae, Phycitinae	80.5	−0.019	19.3	−0.199	[66]
*Nagiella inferior*	NC_040973.1	Crambidae, Spilomelinae	81.5	0.009	18.5	−0.22	Direct submission
*Nomophila noctuella*	NC_025764.1	Crambidae, Spilomelinae	81.4	0.002	18.6	−0.176	[67]
*Omiodes indicata*	NC_039177.1	Crambidae, Spilomelinae	81.6	−0.012	18.4	−0.162	[30]
*Omphisa* *fuscidentalis*	NC_066444.1	Crambidae, Spilomelinae	79	0.013	21	−0.274	Direct submission
*Orthaga euadrusalis*	NC_061246.1	Pyralidae, Epipaschiinae	80.2	−0.02	19.8	−0.199	[54]
**Organism**	**RefSeq ID**	**Taxonomy**	**AT%**	**AT-Skew**	**GC%**	**GC-Skew**	**References**
*Orthaga olivacea*	NC_046504.1	Pyralidae, Epipaschiinae	79	−0.043	21	−0.215	Direct submission
*Orthopygia* *glaucinalis*	NC_047304.1	Pyralidae, Pyralinae	79.2	−0.044	20.8	−0.223	[68]
*Orybina regalis*	NC_061247.1	Pyralidae, Pyralinae	81	−0.016	19	−0.205	[54]
*Ostrinia furnacalis*	NC_056248.1	Crambidae, Pyraustinae	80.9	0.031	19.1	−0.196	[69]
*Ostrinia kasmirica*	NC_059846.1	Crambidae, Pyraustinae	81	0.031	19	−0.192	Direct submission
*Ostrinia nubilalis*	NC_054270.1	Crambidae, Pyraustinae	80.5	0.033	19.4	−0.195	[70]
*Ostrinia scapulalis*	NC_048887.1	Crambidae, Pyraustinae	81	0.03	19.1	−0.196	[51]
*Ostrinia zealis*	NC_048888.1	Crambidae, Pyraustinae	80.9	0.031	19.1	−0.193	[51]
*Palpita hypohomalia*	NC_039632.1	Crambidae, Spilomelinae	81	−0.001	18.9	−0.196	Direct submission
*Paracymoriza* *distinctalis*	NC_023471.1	Crambidae, Acentropinae	82.2	−0.002	17.7	−0.155	[71]
*Paracymoriza* *prodigalis*	NC_020094.1	Crambidae, Acentropinae	81.5	0.002	18.4	−0.183	[72]
*Paralipsa gularis*	NC_054356.1	Pyralidae, Gallerinae	79.5	−0.014	20.5	−0.239	Direct submission
*Parapediasia* *teterrellus*	NC_068594.1	Crambidae, Crambinae	80.5	−0.003	19.5	−0.231	Direct submission
*Parapoynx crisonalis*	NC_031151.1	Crambinade, Acentropinae	82	0.017	18	−0.153	Direct submission
*Perula* sp.	NC_066226.1	Pyralidae, Pyralinae	81	−0.037	19	−0.213	Direct submission
*Plodia interpunctella*	NC_027961.1	Pyralidae, Phycitinae	80.1	−0.05	19.9	−0.233	Direct submission
*Polythlipta liquidalis*	NC_073109.1	Crambidae, Spilomelinae	81	−0.005	19	−0.21	Direct submission
*Prophantis adusta*	NC_067853.1	Crambidae, Spilomelinae	81.5	0.001	18.5	−0.196	Direct submission
*Pseudargyria* *interruptella*	NC_029751.1	Crambidae, Crambinae	79.4	−0.011	20.6	−0.216	Direct submission
*Pseudonoorda* *nigropunctalis*	NC_056801.1	Crambidae, Odontinae	81	−0.003	19	−0.201	[60]
*Pycnarmon* *lactiferalis*	NC_033540.1	Crambidae Spilomelinae	81.7	−0.004	18.3	−0.173	[73]
*Pygospila tyres*	NC_066087.1	Crambidae, Spilomelinae	81.3	−0.008	18.7	−0.158	Direct submission
*Pyrausta despicata*	NC_046050.1	Crambidae, Pyraustinae	80.9	0.009	19	−0.204	Direct submission
*Scirpophaga* *incertulas*	NC_031329.1	Crambidae, Schoenobiinae	77.2	0.029	22.9	−0.32	Direct submission
*Sinomphisa plagialis*	NC_061243.1	Crambidae, Spilomelinae	80.6	−0.008	19.4	−0.216	[54]
*Sitochroa verticalis*	NC_062118.1	Crambidae, Pyraustinae	80.6	0.005	19.5	−0.203	Direct submission
*Syllepte taiwanalis*	NC_061245.1	Crambidae, Pyraustinae	81.7	−0.009	18.3	−0.182	[54]
*Tyspanodes hypsalis*	NC_025569.1	Crambidae, Spilomelinae	81.4	−0.017	18.6	−0.175	[74]
*Tyspanodes striata*	NC_030510.1	Crambidae, Spilomelinae	81.3	−0.018	18.7	−0.177	Direct submission

**Figure 2:**
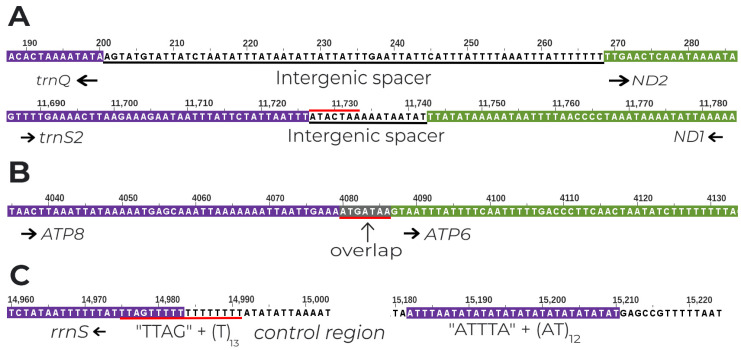
Intergenic spacers, overlapping regions, and conserved motifs in the EFSB mitogenome. (**A**) Intergenic spacers found between *trnQ-ND2* and *trnS2-ND1*. Sequences in black underlines are the spacers (**B**) Gene overlap regions between *ATP8-ATP6*. Sequences highlighted in grey are the overlapping regions. (**C**) Control region motifs found in the EFSB mitogenome. Sequences with the red line indicate conserved motifs, and the arrow direction shows the transcription direction of the genes, which are colored for reference.

The EFSB mitogenome is highly compact, containing mostly genes and the control region with few and small intergenic spacers. The longest spacer is the 69 bp region between *trnQ* and *ND2*. The *trnQ-ND2* spacer is considered to be a feature of lepidopteran mitogenomes [75] and is thought to have arisen from partial duplication of the *ND2* gene [76]. Another spacer in the EFSB mitogenome is the one found between *trnS2* and *nad1*. This spacer is conserved in all insects and contains the motif “ATACTAA” believed to be the recognition site for the mitochondrial transcription termination (mTERM) protein [30,75] (Figure 2A). In addition, overlapping sequences were also found for the EFSB mitogenome. In particular, the 7 bp “ATGATAA” overlap found at the interface of *ATP6* and *ATP8* is another conserved feature of lepidopteran mitogenomes [30,75] (Figure 2B). For the control region, four regions are of interest that are also conserved features of lepidopterans. The “ATTTA” motif followed by a poly-AT stretch of length 12, and the 13 bp poly-T stretch has also been documented in other lepidopterans [30,75,77]. The “ATAG” box near the 5’end of the *s-rna* is believed to be the origin of light strand replication for the mitogenome [53]. Interestingly, for other lepidopteran mitogenomes, the “ATAG” box precedes the poly-T stretch [78,79], but the motif is “TTAG” in EFSB (Figure 2C). 

### 3.2. Protein-Coding Genes

**Figure 3:**
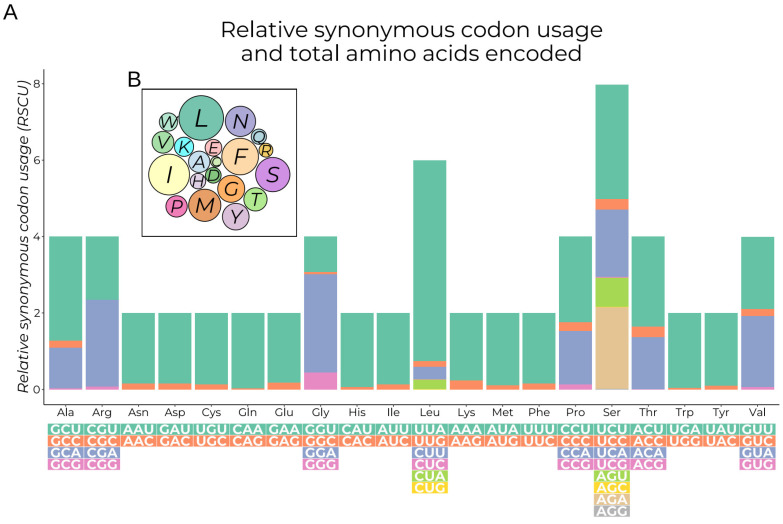
Relative synonymous codon usage and total amino acids encoded by the EFSB mitogenome. (**A**) Relative synonymous codon usage (RSCU) is a measure of the deviation of each codon from the assumption that all codons are used equally. The bar graph shows the relative distribution of codon usage across all amino acid codons. The number of codons for each amino acid ranges from two to eight. The RSCU values are color-coded based on the codons below the amino acid labels. (**B**) Amino acids encoded by the EFSB mitogenome. Each amino acid is colored differently, and the labels in the circles correspond to the one-letter amino acid abbreviations. The number of times a given amino acid was coded is proportional to the area of the circles and the size of the text labels.

The EFSB mitogenome contains 13 protein-coding genes with a total concatenated length of 11,191 bp, comprising 73.41% of the total mitogenome length. All the PCGs are initiated with ATK codons except for *nad2* and *cox1*, which are initiated by TTG codons (Table 2). TTG codons are established initiation codons in invertebrate mitochondria [80], with the TTG codons having relatively high frequencies in the *nad2* and *cox1* genes of invertebrates [81]. With the PCGs having ATK start codons, ATG (7/13) is slightly preferred over ATT (4/13) codons (Table 2). On the other hand, some of the PCGs of the EFSB mitogenome have incomplete stop codons. The genes *COX1*, *ND5*, and *CYTB* have non-canonical T stop codons, while *ATP6* and *ND4* have non-canonical TA (Table 2). Interestingly, both MITOS and MitoZ have annotated canonical TAA stop codons for all the PCGs in the EFSB mitogenomes. However, significant overlap of the annotated stop codons with downstream genes has been observed for some PCGs. Thus, manual checking of the annotated PCG gene boundaries was performed to determine if the gene boundaries were consistent with downstream genes. The presence of incomplete stop codons has been documented for invertebrate mitogenomes and are processed into full TAA stop codons post transcriptionally [82,83,84]. This observation is supported by the analysis of Donath et al. (2019), where the authors show that invertebrate *COX1*, *ND5*, *CYTB*, *ATP6*, and *ND4* have significant frequencies of incomplete stop codons [81]. After manual checking, the gene boundaries of the aforementioned genes were adjusted to end in incomplete stop codons. Total amino acids encoded shows that the EFSB mitochondrion codes for a higher proportion of hydrophobic amino acids, with leucine (L), isoleucine (I), and phenylalanine (F) representing three of the top four amino acids used (Figure 3B). This is expected since most of the proteins encoded by mitogenome are components of membrane-bound, hydrophobic complexes important for energy generation [85]. The relative synonymous codon usage is shown in Figure 3A. RSCU analysis indicates that AT rich codons are preferentially used compared to GC rich codons. For example, the EFSB mitochondrion prefers to utilize the codons UUA, AAU, CAA, and AUU to code for the amino acids leucine (Leu), asparagine (Asn), glutamine (Gln), and isoleucine (Ile), respectively, compared to their counterpart codons. The high AT content of the EFSB mitogenome (Table 1) may be correlated to the bias of using AT-rich codons. Overall, the patterns in codon usage are similar to those observed in other members of Pyraloidea [51,86]. 

### 3.3. Ribosomal and Transfer RNA Genes

**Figure 4:**
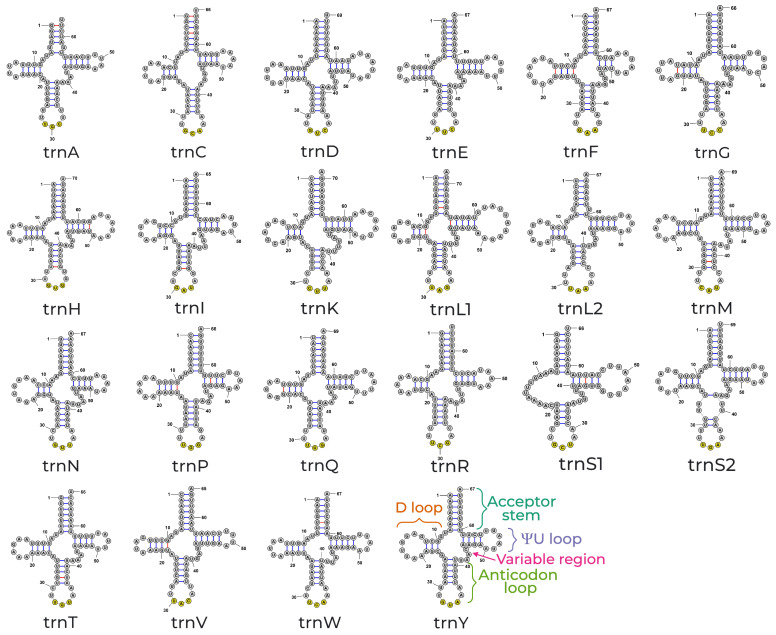
Structures of the EFSB mitogenome tRNAs. All of the EFSB mitogenome tRNAs except for *trnS1* follow the canonical conserved 3-loop cloverleaf structure consisting of the acceptor stem (dark green), dihydrouridine loop (D loop, orange), the pseudouridine arm (ΨU loop, purple), and the anticodon loop (light green). All tRNAs also contain a variable region (pink). In the case of *trnS1*, the D loop fails to form a stem–loop structure. The anticodon for each tRNA is indicated in yellow, while all non-canonical base pairs are indicated by a red bond. All tRNA structures of the EFSB mitogenome were predicted using MITOS, validated using tRNAScan-SE, and drawn using StructureEditor.

The tRNA genes have a total concatenated length of 1479 bp, contributing 9.7% of the total EFSB mitogenome while the rRNA genes have a total length of 2167 bp, contributing 14.21% of the mitogenome. There are 22 tRNA genes, with serine and leucine having two isoacceptors, while the other 18 amino acids have 1. All the tRNAs have been predicted and verified by MITOS [33] and tRNAScanSE [34] except for trnS2, which yielded no homologous search using tRNAScanSE. This may be due to the four unique unpaired uracils in the anticodon loop (Figure 4). However, such a case of four unpaired uracils was also demonstrated in the *trnS2* of the hemp borer, *Grapholita delineana* [87], the *trnS2* of *Plodia interpunctella* [86], and the *trnS2* of five skippers [53]. The length of the tRNAs ranges from 64 bp (*trnA* and *trnR*) to 72 bp (*trnL1*) (Table 2). The secondary structure of tRNAs is generally conserved, forming the typical three-loop, one-stem cloverleaf structures consisting of an amino acid acceptor stem, a dihydrouridine loop (D loop), a pseudouridine loop (ΨU loop), and an anticodon loop. tRNAs also contain a variable region between the anticodon loop and the ΨU loop. All EFSB mitochondrial tRNAs exhibited this typical cloverleaf, except for *trnS1*, where the D loop fails to form a canonical stem–loop structure (Figure 4). The noncanonical structure of the D-armless *trnS1* is a common and conserved feature of metazoan mitogenomes [88]. All the tRNAs have a structurally conserved 7 bp amino acid acceptor stem, except for *trnA* which contains a 6 bp stem. The tRNAs *trnA* and *trnL2* also contain a unique unpaired uracil in the acceptor stem (Figure 4). Similarly, all the tRNAs contain a conserved 5 bp stem, and a 7 bp loop in the anticodon loop, except for *trnL2*, which contains a 4 bp stem, and a 9 bp loop; *trnK* and *trnS1* have a 4 bp stem with a 7 bp loop; and *trnS2* contains a unique anticodon loop with four unpaired uracils (Figure 4). Noncanonical base pairings also occur in the EFSB mitochondrial tRNAs, with G-U pairs comprising all such pairings (Figure 4). Despite the conservation of both the acceptor stem and anticodon loop, the D and ΨU loops show diversity in the number of stem pairings and the number of nucleotides contained in the hairpin loops. Variability in the D and ΨU may indicate slight deviations from the canonical D/T loop interactions important for aminoacylation [89], but are still functional. The EFSB mitogenome contains two ribosomal RNA genes, *l-rna* and *s-rna*. The *l-rna* and *s-rna* genes are 1358 bp- and 809 bp-long, respectively. The length of the EFSB mitogenome rRNA genes is similar to that of other pyraloid moths [30].

### 3.4. Phylogenetic Relationships

A whole mitogenome phylogenetic analysis was used to determine the placement of EFSB within Pyraloidea. The organisms, taxonomy, and reference sequence ID of the mitochondrial genomes used for the analysis are shown in Table 1. For maximum likelihood (ML) and Bayesian inference (BI) analyses, ModelFinder was first used to determine the best-of-fit models for the dataset [47]. For the ML analysis, the initial 37 partitions were reduced to 9 partitions, as summarized in Table S1. ModelFinder found that the general time reversible model (GTR) is the most suitable model for all partitions except partition 3, where the TPM2 model is the most suitable. The GTR model is the model where all six substitution rates and all four base frequencies are unequal. The TPM2 model, on the other hand, assumes that the base frequencies are equal, and the substitution rates have the following relationship: AC=AT, AG=CT, and CG=GT [45]. A similar case was observed when applying ModelFinder for the BI analysis. The initial 37 partitions were also reduced to 9, and ModelFinder found that the GTR model is the most suitable for all partitions for BI. In addition, the corresponding parameters for the best model for each partition were used as the priors for the BI run. All the parameters used as priors are summarized in Table S2. Upon assessing the individual MCMC runs, all three runs converged, as evidenced by the similarly shaped violin plots of the total branch length parameter (Figure S3A). The trace plot for the total branch length across all generations also showed a fairly constant value with small fluctuations around the mean. Lastly, the diagnostic parameters average effective sample size (ESS) and the potential scale reduction factor (PSRF) for the total branch length are 6742.56 and 1.000, respectively (Table S3), indicating that the three MCMC runs converged. 

**Figure 5:**
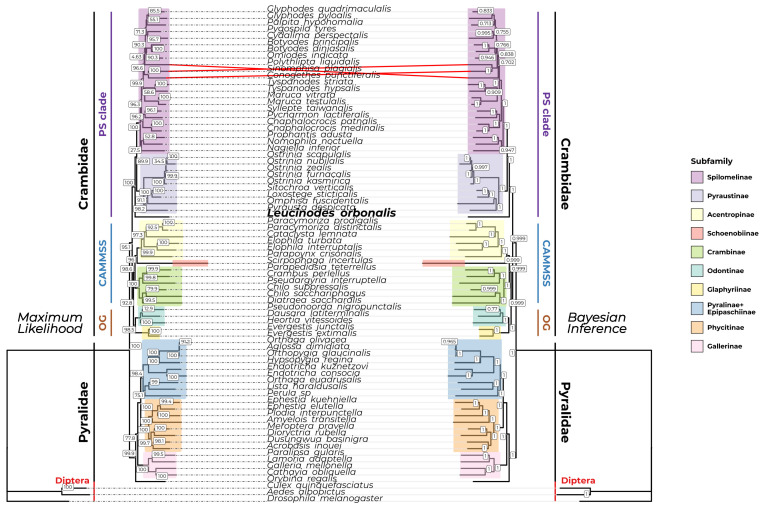
Topological concordance between the partitioned maximum likelihood and Bayesian inference phylogenetic trees. The maximum likelihood (ML) and Bayesian inference (BI) phylogenetic trees are shown on the left- and right-hand side of the labels, respectively. The labeling scheme follows the tip order from the ML tree as indicated by the dotted lines. The subfamilies are coded according to color. The grey lines connecting the tips of both trees indicate tips that agree for both trees, while the red lines indicate the tips with a different order and topology between the trees. SH_aLRT and posterior probability support values are shown for the ML and BI trees, respectively. The subfamilies included in the analysis are Spilomelinae, Pyraustinae, Acentropinae, Schoenobiinae, Crambinae, Odontinae, and Glaphyriinae of Crambidae; and Gallerinae, Phycitinae, Pyralinae, and Epipaschiinae of Pyralidae. The outgroups chosen for the analysis consists of the dipterans *Drosophila melanogaster*, *Culex quinquefasciatus*, and *Aedes albopictus*. The subfamilies are highlighted according to color. The EFSB is highlighted in bold for emphasis. The PS (Pyraustinae-Spilomelinae), CAMMSS (Crambinae, Acentropinae, Midilinae, Musotiminae, Schoenobiinae, and Scopariinae), and OG (Odontinae-Glaphyriinae) clades are the clades first defined by Regier et al. (2012) [16].

The side-by-side comparison of the obtained phylogenetic trees is shown in Figure 5, while the consensus ML and BI trees are shown in Figure S4. The ML and BI trees are mostly concordant with each other. The general subfamily relationships within the Pyraloidea agree for both trees, with Pyraloidea having the basal split to Crambidae and Pyralidae. The general relationships across Pyralidae also agree with both trees, having recovered the (Gallerinae, (Phycitinae, (Pyralinae, Epipaschiinae))) relationships. Similarly, the ((Spilomelinae, Pyraustinae), ((Crambinae, (Schoenobiinae, Acentropinae)), (Odontinae, Glaphyriinae))) relationships in Crambidae were recovered for both trees (Figure 5). The only disagreements between the ML and BI trees were the order of emergence between *Polythlipta liquidalis*, *Sinomphisa plagialis*, and *Conogethes punctiferalis*. For the ML tree, the clade containing *S. plagialis* and *C. punctiferalis* is basal to *P. liquidalis*, but the BI tree shows that *C. punctiferalis* is basal to the clade containing *P. liquidalis* and *S. plagialis* (Figure 5). Nevertheless, on all other tips, the two trees agree.

The root node for Pyraloidea is highly supported (100% bootstrap support (BS); 1 posterior probability (PP)), indicating the monophyly and shared ancestry for this superfamily. The expected basal split and monophyly of both Crambidae and Pyralidae were recovered, as shown in Figure 5 and Figure S4. The split is highly supported (95.1% BS; 0.999 PP), concordant with the earlier works of Regier et al. (2012) [16] and Léger et al. (2020) [17]. The relationships between the four subfamilies of Pyralidae show high support values. A split in the clade consisting of Gallerinae and the clade consisting of Phycitinae, Pyralinae, and Epipaschiinae is moderately supported in the ML tree (77.8% BS) but well supported in the BI tree (1 PP). Phycitinae was recovered to be a sister clade to the clade containing Pyralinae and Epipaschiinae, and this is also highly supported (100% BS; 1 PP) and consistent with earlier works [17,54,62]. Interestingly, the analysis failed to resolve the subfamily relationships between Pyralinae and Epipaschiinae, exemplified by *Orthaga olivacea*, which is classified under Epipaschiinae, showing closer relationships to *Aglossa dimidiata* than *Lista haraldusalis*, which are Pyralinae and Epipaschiinae, respectively. Similarly, *Perula* sp., which is Pyralinae, clusters closer to *Lista haraldusalis* than to other Pyralinae. The relationships between the subfamilies of Crambidae also show relatively good bootstrap support values. Crambidae was recovered to be monophyletic and showed the split between the clade consisting of Pyraustinae and Spilomelinae (PS clade) and the clade containing all other subfamilies (non-PS clade). This expected split is highly supported (95.1% BS; 0.999 PP). The non-PS clade shows a split between the Odontinae-Glaphyriinae (OG) clade and the Crambinae, Acentropinae, Midilinae, Musotiminae, Schoenobiinae, and Scopariinae (CAMMSS) clade, and this split is well-supported (92.8% BS; 0.999 BI). The sister relationships between CAMMSS and OG, and Odontinae and Glaphyriinae, are consistent with the findings of Qi et al. (2021) [60]. In the CAMMSS, Schoenobiinae was recovered to be a sister to Acentropinae. This result is consistent with earlier mitogenomic works [54,90], but different from others [60,62], which places Schoenobiinae as a sister to Crambinae. The subfamilies represented in the non-PS clade were recovered to be monophyletic, and this is well supported for in both trees (Glaphyriinae: 100% BS; 1 PP, Odontinae: 100% BS; 1 PP, Crambinae: 100% BS, 1 PP, Acentropinae: 99.9% BS; 1 PP) (Figure 5).

The general subfamily relationships in Pyraloidea obtained in Figure 5 and Figure S4 are consistent with earlier works [16,17]. Interestingly, while Pyraustinae was recovered to be monophyletic (100% BS; 1 PP), Spilomelinae was recovered to be paraphyletic. The EFSB is traditionally classified under Spilomelinae, but both ML and BI trees show that EFSB emerged before the split of Pyraustinae and Spilomelinae (Figure 5). The placement of EFSB within the PS clade is highly supported (100% BS; 1 PP). Spilomelinae without EFSB is also highly supported (100% BS; 1 PP), indicating that EFSB may have emerged before the PS split. Such a case was also documented for the pyralid moth *Orybina regalis*. Traditionally placed within Pyralinae, *Orybina regalis* was recovered to be basal to Gallerinae in the analysis by Liu et al. (2021) [54]. The analysis conducted here also recovered *O. regalis* to be basal to Gallerinae in both ML and BI trees (Figure 5 and Figure S4). Additionally, while the subfamily relationships recovered in this study are consistent with earlier works, the lower-level relationships vary. There are some species which are robustly placed in other subfamilies. For example, *Omphisa fuscidentalis* is classified under Spilomelinae but is robustly placed in Pyraustinae in both ML and BI trees (Figure 5 and Figure S4). A similar case was observed for *Syllepte taiwanalis* and both *Cnaphalocrocis* species. Both are classified under Pyraustinae but were robustly placed in Spilomelinae. It is interesting to note, however, that such placement for the said taxa was also observed in earlier works [54,90]. The earlier work by Tang and Du (2023) shows consistent subfamily relationships with those obtained in Figure 5 but differ greatly in the lower-level relationships within Spilomelinae [90]. In Figure 5, the tribe Spilomelini is a sister to the clade containing *Pycnarmon lactiferalis* and *Syllepte taiwanalis*, and their collective clade is a sister to the tribe Margaroniini, which in Figure 5 is the clade containing the genus *Glyphodes* and *Maruca*. This relationship is concordant with Tang and Du (2023) [90]. However, the relationships within Margaroniini are different. For example, *Palpita hypohomalia* has close relationships with the genus *Glyphodes* in Figure 5, but the same species has a closer relationship with *Cydalima perspectalis* in Tang and Du (2023) [90]. Additionally, while the sister relationships between the tribes Trichaeini (*Prophantis adusta*) and Nomophilini (*Nomophila noctuella)* can be observed, *Nagiella inferior* clusters close to these two, which is different from the relationship observed in Tang and Du (2023) [90].

Based on the above analyses, the phylogenetic trees obtained in this work are largely consistent with earlier works. Both ML and BI trees robustly place *Leucinodes orbonalis* in the PS clade but are basal to the split of Pyraustinae and Spilomelinae. However, there is still the possibility of long-branch attraction. The incorporation of more mitochondrial sequences from Pyraloidea and the incorporation of nuclear genes may be able to resolve the lower-level relationships with Spilomelinae and produce a more conclusive phylogenetic analysis for this economically important subfamily.

## 4. Summary and Conclusions

The whole mitochondrial genome of the eggplant fruit and shoot borer was assembled and annotated in this study, producing a complete, circular 15,244 bp-long mitogenome that contains the 37 genes expected for a pyraloid mitochondrion. The EFSB mitogenome contains conserved lepidopteran mitogenome features such as the control region motifs, and the *trnM-trnI-trnQ-nad2* synteny. Partitioned phylogenetic analysis using the EFSB sequence assembled in this study recovered the expected basal split of the families Crambidae and Pyralidae and the split between the subfamilies Spilomelinae and Pyraustinae. However, Spilomelinae was recovered to be paraphyletic, as indicated by the robust placing of EFSB before the split of Spilomelinae and Pyraustinae in both ML and BI trees. The addition of more mitochondrial sequences from Pyraloidea and the incorporation of nuclear genes should yield a more resolved phylogeny that details the genus-level relationships within the economically important Spilomelinae. Overall, the mitogenome produced here contributes to the scarce genetic resources for the EFSB and will be a great help in future phylogenetic reconstruction of Spilomelinae and in future population studies and studies that will impact EFSB management.

## Figures and Tables

**Table 2:** Summary of the genes for the *Leucinodes orbonalis* Guenée mitogenome.

Gene	Location	Strand	Start Codon	Stop Codon	Length (bp)	Anticodon
*trnM*	1–69	Majority	-	-	69	CAU
*trnI*	70–134	Majority	-	-	65	GAU
*trnQ*	132–200	Minority	-	-	69	UUG
*ND2*	269–1270	Majority	TTG	TAA	1002	-
*trnW*	1285–1351	Majority	-	-	67	UCA
*trnC*	1344–1409	Minority	-	-	66	GCA
*trnY*	1413–1479	Minority	-	-	67	GUA
*COX1*	1493–3026	Majority	TTG	T	1534	-
*trnL2*	3027–3093	Majority	-	-	67	UAA
*COX2*	3094–3774	Majority	ATG	TAA	681	-
*trnK*	3779–3849	Majority	-	-	71	CUU
*trnD*	3850–3918	Majority	-	-	69	GUC
*ATP8*	3919–4086	Majority	ATT	TAA	168	-
*ATP6*	4080–4759	Majority	ATG	TA	680	-
*COX3*	4760–5548	Majority	ATG	TAA	789	-
*trnG*	5551–5616	Majority	-	-	66	UCC
*ND3*	5617–5970	Majority	ATT	TAA	354	-
*trnA*	5975–6038	Majority	-	-	64	UGC
*trnR*	6039–6102	Majority	-	-	64	UCG
*trnN*	6102–6168	Majority	-	-	67	GUU
*trnS1*	6171–6236	Majority	-	-	66	GCU
*trnE*	6240–6306	Majority	-	-	67	UUC
*trnF*	6326–6392	Minority	-	-	67	GAA
*ND5*	6393–8124	Minority	ATT	T	1732	-
*trnH*	8125–8194	Minority	-	-	70	GUG
*ND4*	8195–9534	Minority	ATG	TA	1340	-
*ND4L*	9544–9834	Minority	ATG	TAA	291	-
*trnT*	9840–9905	Majority	-	-	66	UGU
*trnP*	9906–9971	Minority	-	-	66	UGG
*ND6*	9974–10507	Majority	ATT	TAA	534	-
*CYTB*	10511–11657	Majority	ATG	T	1147	-
*trnS2*	11658–11726	Majority	-	-	69	UGA
*ND1*	11743–12681	Minority	ATG	TAA	939	-
*trnL1*	12683–12754	Minority	-	-	72	UAG
*l-rna*	12764–14121	Minority	-	-	1358	-
*trnV*	14110–14174	Minority	-	-	65	UAC
*s-rna*	14175–14983	Minority	-	-	809	-

## Data Availability

The sequence of the final mitogenome assembly has been deposited into GenBank under accession number PP493058. The final mitogenome assembly, intermediate files, and scripts used for the analysis of the mitogenome are deposited and openly available in Zenodo at 10.5281/zenodo.10653202.

## Supplementary Materials

The following supporting information can be downloaded at: https://www.mdpi.com/article/10.3390/insects15040220/s1, Figure S1. Sequence quality of the EFSB mitogenome reads; Figure S2. Cumulative coverage distribution for the EFSB mitogenome; Figure S3. Estimation of the total branch length parameter across 3 MCMC runs; Figure S4. Partitioned phylogenetic analysis of 72 members of the superfamily Pyraloidea and 3 dipterans using maximum likelihood and Bayesian inference; Table S1. Final gene partitions for the Maximum Likelihood Phylogenetic analysis; Table S2. Final gene partitions for the Bayesian Inference Phylogenetic analysis; Table S3. 95% Credibility interval of the sampled parameters during the MCMC run.
