# Supplementary material for: Complete Mitochondrial Genome of the Eggplant Fruit and Shoot Borer, Leucinodes orbonalis Guenée (Lepidoptera: Crambidae), and Comparison with Other Pyraloid Moths
Source: Insects. 2024 Mar 25;15(4):220. doi: 10.3390/insects15040220 (PMC11050083; doi:10.3390/insects15040220)
Supplement: Supplementary file 1 [file insects-15-00220-s001.zip › insects-2900429-supplementary.pdf]

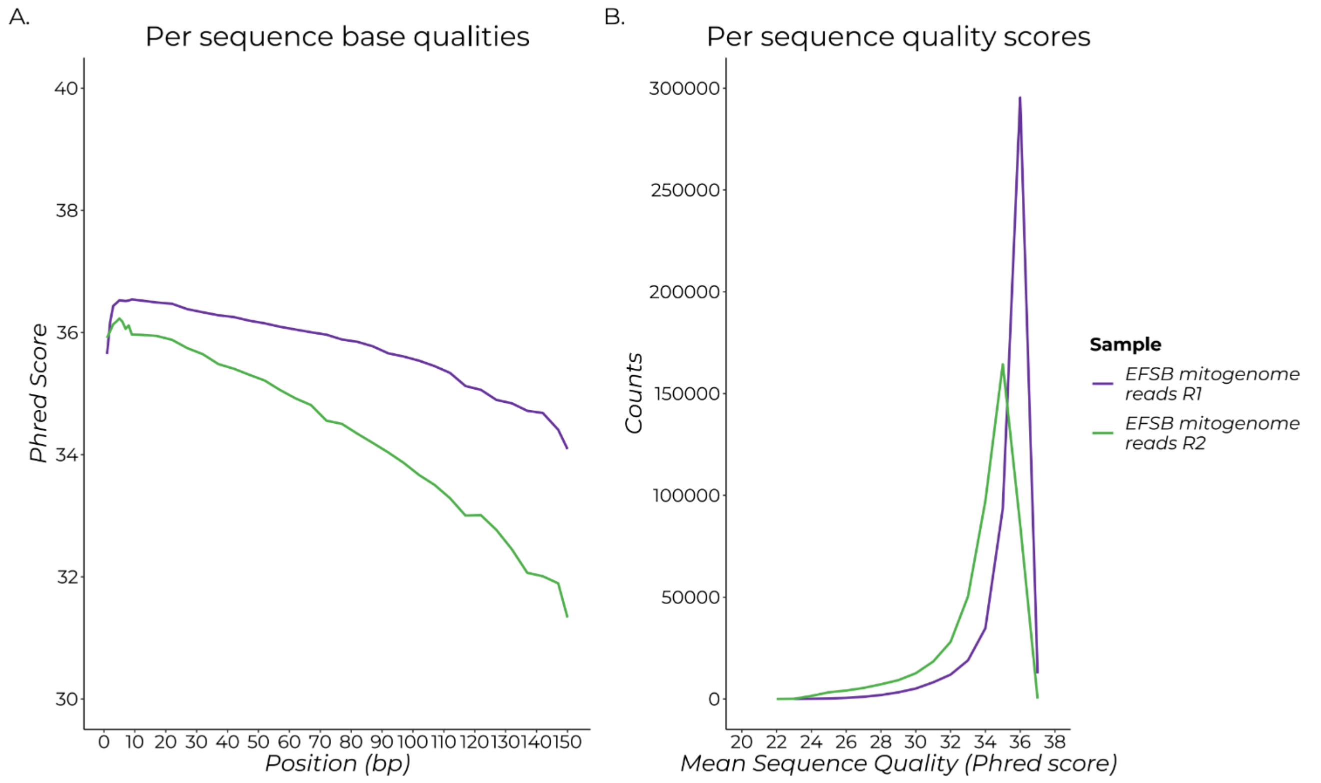

Figure S1. Sequence quality of the EFSB mitogenome reads. (A). Mean sequence qualities per base of all sequencing reads. (B). Distribution of sequencing reads according to mean sequence quality (Phred score). Lines in purple for both plots show the qualities of the forward (R1) reads, and the lines in green show the qualities of the reverse (R2) reads.

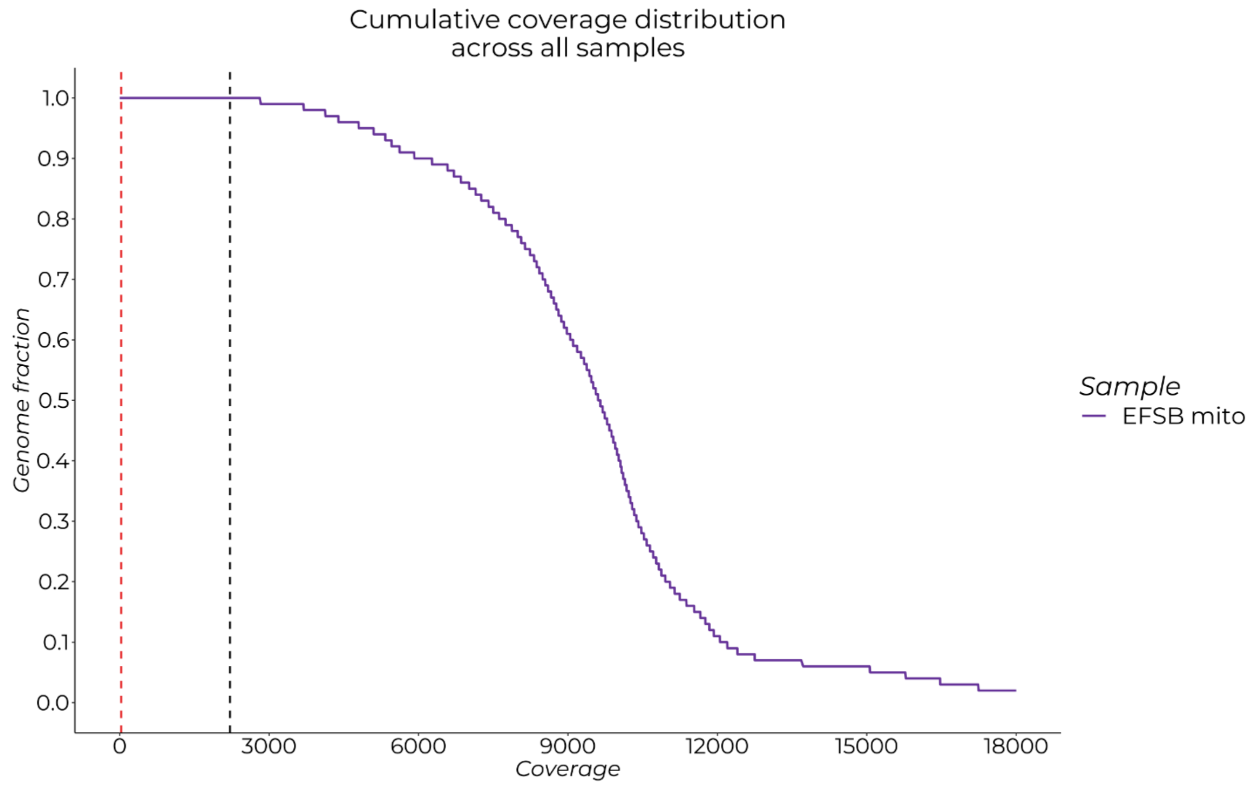

Figure S2. Cumulative coverage distribution for the EFSB mitogenome. The plot shows the coverage distribution plotted against genome fraction. The dashed line in red indicates 30X coverage, while the line in black indicates 2215X coverage.

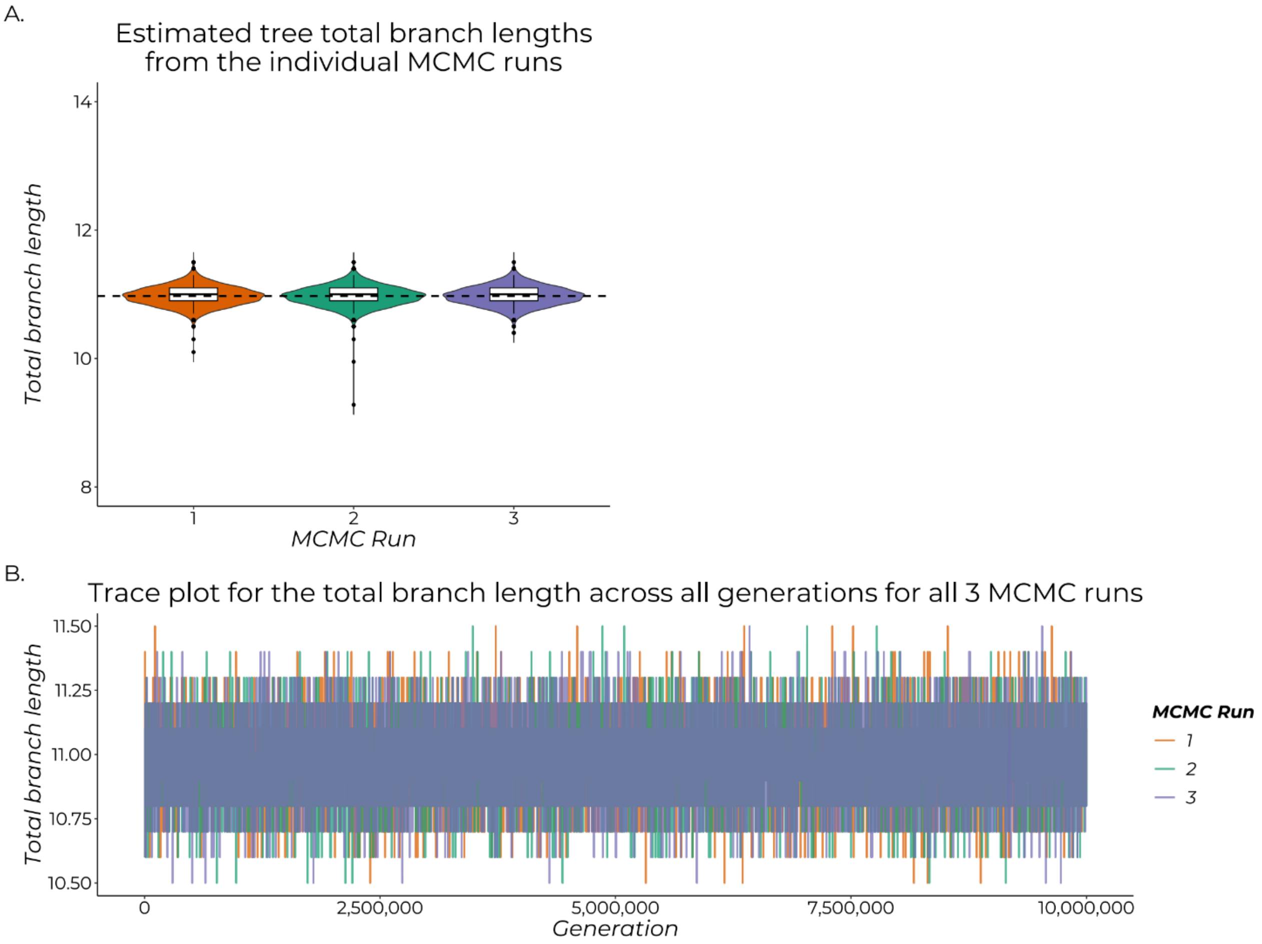

Figure S3. Estimation of the total branch length parameter across 3 MCMC runs. (A). The violin plot shows the distribution of the total branch length values across 10,000,000 generations of 3 MCMC runs. The violin plots are colored according to the run, and boxplots are shown inside the violin to show the mean total branch length value for each run. The dashed black line corresponds to the mean total branch length value (10.973) obtained across all three runs for reference. (B). The plot shows the trace diagram of each MCMC run, showing the obtained total branch length values per generation. The plot shows the values are clustered close to the mean total branch length value (10.973) with slight deviations, forming the hairy caterpillar shape of the trace plot indicative of chain convergence.

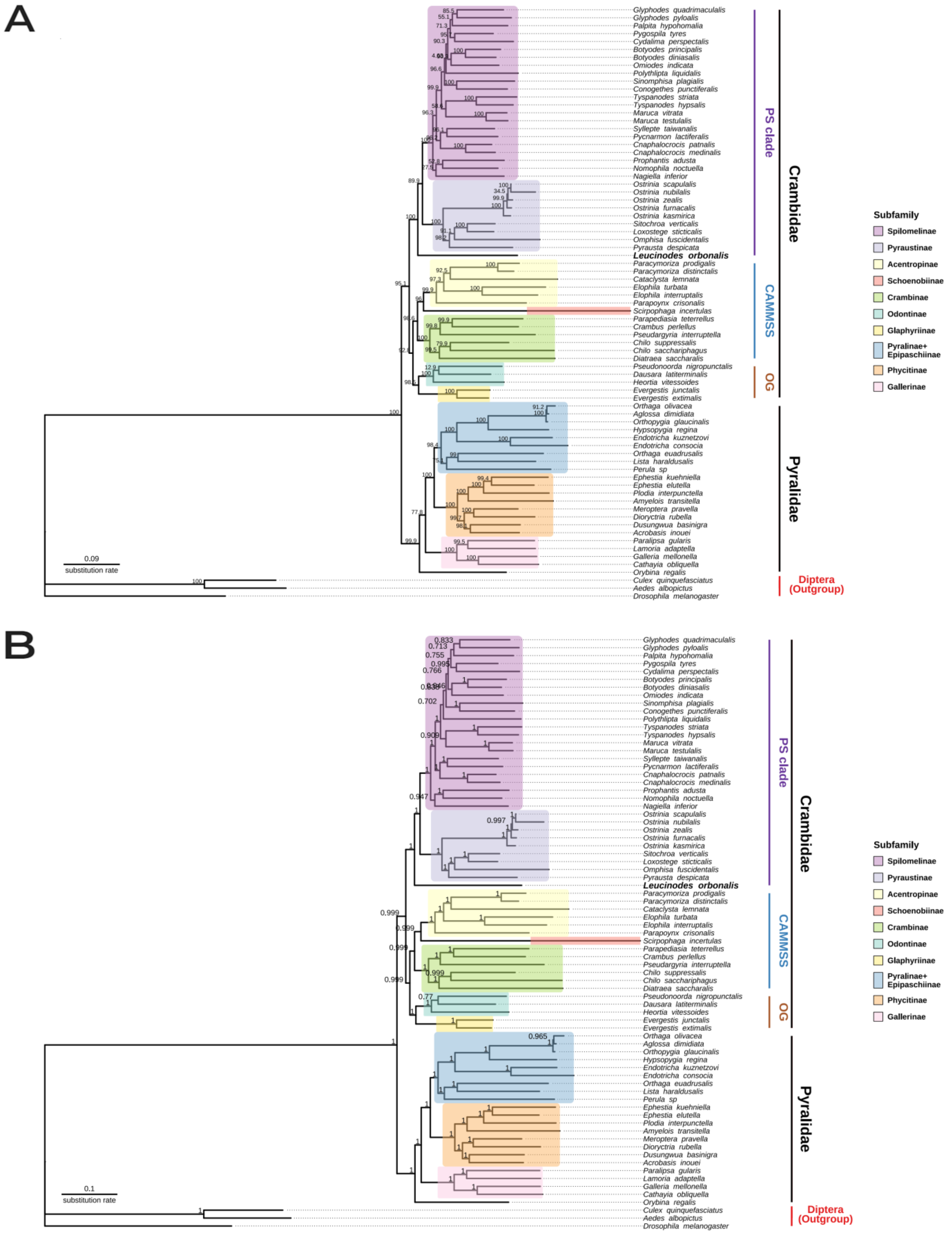

Figure S4. Partitioned phylogenetic analysis of 72 members of the superfamily Pyraloidea and 3 dipterans using maximum likelihood and Bayesian inference. Pyraloidea is comprised of mostly two families, Crambidae and Pyralidae. Both families are further divided into subfamilies, and the representative subfamilies included in the analysis consist of Spilomelinae, Pyraustinae, Acentropinae, Schoenobiinae, Crambinae, Odontinae and Glaphyriinae of Crambidae; and Gallerinae, Phycitinae, Pyralinae and Epipaschiinae of Pyralidae. The outgroups chosen for the analysis consist of the dipterans *Drosophila melanogaster*, *Culex quinquefasciatus*, and *Aedes albopictus*.

The subfamilies are highlighted according to color. The EFSB is highlighted in bold for emphasis. (A) The numbers shown in each node are SH-aLRT bootstrap support values calculated by IQ-tree using 10,000 replicates, and the branch lengths are the number of nucleotide substitutions per site. (B) The numbers shown in each node are the posterior probabilities for the node calculated by MrBayes across 10,000,000 generations. The PS (Pyraustinae-Spilomelinae), CAMMSS (Crambinae, Acentropinae, Midilinae, Musotiminae, Schoenobiinae, and Scopariinae) and OG (Odontinae-Glaphyriinae) clades are the clades first defined by Regier et al. (2012).

Table S1. Final gene partitions for the Maximum Likelihood Phylogenetic analysis

Partition number	Included genes/regions	Substitution Model	Log likelihood	BIC score[a]
1	*cox1, trnK, trnM*	GTR+F+R5	-30275.56	60677.199
2	*nad1, nad4, nad5, nad4L*	GTR+F+R6	-91033.13	182225.212
3	*trnL1, trnS1, trnL2, trnC, trnF, trnI, trnQ, trnY*	TPM2+F+R4	-5292.456	10657.734
4	*cox2, cox3, cytb, trnE*	GTR+F+R6	-54989.432	110128.722
5	*nad2*	GTR+F+R5	-24715.833	49549.115
6	*trnS2, trnA, trnD, trnN, trnR, trnT, trnV, trnW*	GTR+F+R4	-5404.585	10900.975
7	*nad3, atp6*	GTR+F+R5	-23399.183	46916.267
8	*nad6, atp8*	GTR+F+R5	-20139.610	40390.194
9	*rrnL, rrnS, trnG, trnH, trnP*	GTR+F+R4	-25400.362	50912.554

[a] BIC denotes the Bayesian Information Criterion used for model selection

Table S2. Final gene partitions for the Bayesian Inference Phylogenetic analysis

Partition number	Genes	Substitution Model	Log likelihood	BIC score [a]	Substitution rates [b]	Nucleotide frequencies [c]	Proportion of Invariant Sites [d]	Shape parameter [e]
1	*cox1, trnK, trnM*	GTR+F+I+G4	-30321.565	60724.711	(6.28376, 15.852, 26.7175, 8.44516, 100)	(0.317704, 0.145513, 0.137862, 0.398921)	0.436778	0.554488
2	*nad1, nad4, nad5, nad4L*	GTR+F+I+G4	-91334.556	182761.137	(0.303599, 8.72597, 2.57917, 4.3482, 3.09324)	(0.332083, 0.0652726, 0.124446, 0.478198)	0.278017	0.653104
3	*trnL1, trnS1, trnL2, trnC, trnF, trnI, trnQ, trnY*	GTR+F+G4	-5333.337	10727.357	(1.21069, 7.63265, 2.3441, 0.739127, 7.32012)	(0.378767, 0.0779132, 0.142274, 0.401046)	-	0.317339
4	*cox2, cox3, cytb, trnE*	GTR+F+I+G4	-55139.345	110365.449	(6.01147, 16.6387, 15.6029, 4.82504, 100)	(0.339923, 0.134565, 0.107745, 0.417767)	0.351933	0.514513
5	*nad2*	GTR+F+I+G4	-24779.501	49634.998	(2.40168, 7.46581, 3.89376, 6.68363, 17.6029)	(0.350506, 0.0931034, 0.0615972, 0.494793)	0.200061	0.693441
6	*trnS2, trnA, trnD, trnN, trnR, trnT, trnV, trnW*	GTR+F+I+G4	-5440.065	10947.452	(1.50602, 8.67068, 12.628, 0.0563723, 20.6734)	(0.440921, 0.0788901, 0.105373, 0.374816)	0.324024	0.35799
7	*nad3, atp6*	GTR+F+I+G4	-23423.065	46922.419	(3.84597, 12.4008, 10.5787, 8.57317, 41.7035)	(0.337831, 0.128207, 0.0799589, 0.454002)	0.310262	0.664164
8	*nad6, atp8*	GTR+F+I+G4	-20181.494	40434.796	(6.06944, 6.39568, 4.94558, 13.5078, 30.8479)	(0.390519, 0.082746, 0.0466984, 0.480037)	0.119654	0.611777
9	*rrnL, rrnS, trnG, trnH, trnP*	GTR+F+G4	-25647.492	51369.537	(0.787658, 5.91847, 3.38717, 0.337279, 3.68663)	(0.414152, 0.0620746, 0.120684, 0.403089)	-	0.323594

[a] BIC denotes the Bayesian Information Criterion used for model selection

[b]The substitution rates column is defined as the ordered sextuplet of the following nucleotide substitutions: A↔C, A↔G, A↔T, C↔G, C↔T, G↔T. Only 5 values are shown here since by convention, the G↔T rate is set to 1, and the other 5 rates are relative rates.

[c]The nucleotide frequency column is defined as the ordered quadruplet of the frequencies of the nucleotides in the following order: A, C, G, T.

[d] Only the partitions where the substitution model chosen has invariant sites (+I) contain a value for the proportion of invariant sites

[e]The shape parameter column describes the shape parameter of the gamma distribution for each partition.

Table S3. 95% Credibility interval of the sampled parameters during the MCMC run

Parameter	Mean	Variance	Lower bound	Upper bound	Average ESS [a]	PSRF [b]
Total tree length	10.972876	0.019642	10.698820	11.244200	6742.56	1.000

[a] ESS denotes the effective sample size

[b] PSRF denotes the potential scale reduction factor. Both ESS and PSRF are convergence diagnostics used to assess the convergence of independent chains of an MCMC run, where an ESS of greater than 200 and a PSRF of close to 1 being the benchmark values used in determining convergence
